# Detection of a novel porcine parvovirus, PPV4, in chinese swine herds

**DOI:** 10.1186/1743-422X-7-333

**Published:** 2010-11-21

**Authors:** Lv Huang, Shao-Lun Zhai, Andrew K Cheung, Hong-Biao Zhang, Jin-Xue Long, Shi-Shan Yuan

**Affiliations:** 1 Department of Swine Infectious Diseases, Shanghai Veterinary Research Institute, Chinese Academy of Agricultural Sciences, Shanghai 200241, China; 2 College of Veterinary Medicine, Xinjiang Agricultural University, Urumqi 830052, China; 3 Virus and Prion Research Unit, National Animal Disease Center, PO Box 70, Ames, IA 50010, USA; 4 College of Veterinary Medicine, Nanjing Agricultural University, Nanjing 210095, China

## Abstract

To determine whether the novel porcine parvovirus type 4 (PPV4) recently reported in America is prevalent in China, a set of specific primers was designed and used for molecular survey of PPV4 among the clinical samples collected from various provinces of China between 2006 and 2010. The results showed that PPV4 is present in Chinese swine herds at a rate of 2.09% (12/573) among the clinical samples examined and 0.76% (1/132) among the samples taken from healthy animals. We also noted that PPV4 was not detected in samples taken prior to 2009. Analysis of the coding sequences showed that the Chinese and American PPV4 genome sequences are closely related with greater than 99% nucleotide sequence identity. Similar to a previous study, viral genomes in head-to-tail configuration of various lengths of the non-coding region were detected. Our findings confirmed that PPV4 is a unique recently discovered virus in pigs. Phylogenetically, PPV4 is most closely related to bovine parvovirus 2 (BPV2, which is not a *Bocavirus *and is not assigned to any *Parvovirinae *genus) and shares limited ORF1 (33.6%) and ORF2 (24.5%) amino acid identity. With respect to genome structure and organization, PPV4 encodes an ORF3 in the middle of the viral genome that resembles the *Bocavirus *genus. However, the PPV4 ORF3 encoded protein shares minimal amino acid identity with the ORF3 encoded proteins of the *Bocavirus *genus.

## Findings

The International Committee on Taxonomy of Viruses has classified the *Parvovirinae *subfamily of viruses into five genera: *Dependovirus*, *Bocavirus*, *Erythrovirus*, *Parvovirus *and *Amdovirus*. Recently, two new genera, *Hokovirus and Cnvirus*, have been proposed. *Hokovirus *includes a group of porcine and bovine isolates that clustered with the human PARV4 and PARV5 viruses [1,2] and *Cnvirus *includes the H-1 isolate from Myanmar [3] and a group of newly identified porcine isolates from China that exhibit a distinct sublineage from the *Hokovirus *[4,5]. In addition, there are two bovine isolates (BPV2 and BPV3) and one porcine isolate (PPV4) that do not cluster with any member of the *Parvovirinae *genera [6]. The genomes of the *Parvovirinae *members are linear single-stranded DNA of about 5 kilobases that contain terminal palindromic sequences. In general, the genome encodes two major open reading frames (ORFs) coding for the non-structural protein(s) located at the 5'-end and the capsid protein(s) located at the 3'-end. An additional ORF3 located in the middle of the viral genome was observed among viruses of the *Bocavirus *genus (bovine parvovirus [BPV1], canine minute virus [MVC], human bocavirus [HBoV], and, potentially, a partially sequenced porcine boca-like virus [Pbo-likeV]) [7,8]. PPV4 is unique in that its genome nucleotide sequence is most related to BPV2, but the coding capacity and genome organization are more related to viruses of the *Bocavirus *genus [6]. Limited amino acid identity, ORF1 (33.6%) and ORF2 (24.5%), exists between PPV4 and BPV2. The ORF3 amino acid identity among the three recognized *Bocavirus *members is 43.3-47.0%. Whereas the Pbo-likeV ORF3 shares 33.6-35.6% amino acid identity with the recognized *Bocaviruses*, the PPV4 ORF3 shares only 4.9-11.2% [6-8]. The ORF3 of Pbo-likeV and PPV4 share low nucleotide identity (45%) and amino acid identity (17.5%), which indicate Pbo-likeV and PPV4 are two distinct parvoviruses.

The aim of this study was to investigate whether PPV4 exists among swine herds in China by a PCR assay. A total of 705 animals were examined. A sample was collected from each pig (573 sick animals and 132 healthy animals) in 12 provinces of China from 2006 to 2010(100 samples in 2006, 100 samples in 2007,100 samples in 2008,281 samples in 2009,124 samples in 2010). The sick pigs exhibited clinical symptoms that encompassed trembling, fever, testicular atrophy, abortion and death. The samples included 386 sera and 319 tissues from various organs. Briefly, viral DNA was extracted using a DNA/RNA extraction kit (catalog # DP315, Tiangen Biotech Inc., Beijing, China). Based on the PPV4 genomic sequence (GenBank accession numbers: GQ387499) [9], a pair of detection primers [PPV4-F (5'-GGGCGAGAACATTGAAGAGGT-3') and PPV4-R (5'-TTGTGAGTATGGGTATTGTGT-3')] targeting a 543 nucleotide sequence within ORF2 were used. The PCR reaction was performed according to manufacturer's instruction (catalog # KT201-2, Tiangen Biotech Inc., China). The cycling program was as follows: pre-denaturation at 95°C for 5 min, 40 cycles of 95°C for 30 s, 53°C for 30 s and 72°C for 40 s, final extension for 10 min at 72°C. The PCR products were gel-purified and cloned into the PCR2.1T/A vector (Invitrogen Corporation, California, U.S.A.). Nucleotide sequences were determined using an AB-3730 automated DNA sequencer and the results obtained were analyzed with the BLASTx program http://www.ncbi.nlm.nih.gov/blast/. The results showed that 12 out of 573 clinical samples and 1 out of 132 healthy pig samples were positive for PPV4. PPV4-positive pigs were not detected among samples collected from 2006-2008. Eight of the positive samples were collected in 2009 and the other five were collected in 2010 and they came from five different breeding pig farms located in three provinces of China. Although the overall detection rate was low (13/705 = 1.84%), the rate in each positive farm was high: 37% (7/19), 25% (1/4), 40% (2/5), 20% (2/10) and 50% (1/2). Among the thirteen PPV4-positive samples identified, 10 samples were from adult pigs (7 sows about seven months and 3 boars about four months old), two from a dead piglet (about 8 weeks old) and one from a healthy piglet (about 4 weeks old). The adult pigs were diagnosed with reproductive failure, while the sick piglet had displayed fever and neurologic symptoms. Further analysis showed that PPV4 DNA existed in the heart, serum, lymph node, lung and kidney, with the heart containing the highest viral load (data not shown).

To explore whether there were any co-infecting viruses present in our PPV4-positive pigs, we investigated the presence of porcine reproductive and respiratory syndrome virus (PRRSV), porcine circovirus 2 (PCV2), classic swine fever (CSFV), PPV1, PPV2, PPV3, pseudorabies virus (PrV) and porcine torque teno virus (genogroups 1 [PTTV1] and 2 [PTTV2]) according to the protocols previously described [1,3,9-11]. The results revealed that all 13 PPV4-positive samples were co-infected with both PTTV1 and PTTV2, while 4 out of 13 samples (30.7%) were co-infected with PCV2. The other viruses examined were not detected.

Three PPV4-positive pigs (2 sows and 1 boar) from two farms in two provinces: Jiangsu (JS0910 and JS0918, from the same farm) and Henan (HEN0922) were selected for full-length genome sequence determination. Seven pairs of primers (Table 1) (based on of GenBank sequence GQ387500) [6] were designed and used to determine the coding sequences, in the 5' to 3' orientation, from the beginning of ORF1, through ORF3, to the end of ORF2. The nucleotide sequences obtained were assembled using the Seqman software and aligned using CLUSTALW of alignment software and a phylogenetic tree was generated using MEGA 4.1 software [12] and the neighbor-joining method [13].

**Table 1:** Primers for the amplification of the full-length genome sequence of PPV4.

#	Primers	Sequences	Position*	Tm (°C)
1	PF356	5' TGACGCAGTACAGACCGACGAGA 3'	356	60
	PR1197	5' AATGCAAGTGCAAGCCACCTTTT 3'	1197	
2	PF1050	5' AGTAATCTGGTAATCGCTGTTCG 3'	1050	
	PR1942	5' ATGTTAGTCTTTCCTGTTGTGGC 3'	1942	
3	PF1568	5' GCTGGTGGATAACAACATCTGCT 3'	1568	
	PR2553	5' GTTTCTTCTTTCTCGGTGCTTCT 3'	2553	
4	PF2530	5' AAGAAGCACCGAGAAAGAAGAAA 3'	2530	
	PR3413	5' AAATCTAAGGGACAAGGCAAACG 3'	3413	
5	PF3363	5' AGATACTAAGAAAGACAAGGTGGAG 3'	3363	
	PR4263	5' AATAATAGAAGGTATAGCGTCTCCA 3'	4263	
6	PF3918	5' ACCTGCTCCTCCATCTTCTCCAC 3'	3918	
	PR4897	5' GGCCGTCATCATACATTCTGCTC 3'	4897	
7	PF4219	5' ACTTACTGTTCTATGATGTCTGGAG 3'	4219	
	PR5862	5' ATATCATCTGCGGTGTCTGGG 3'	5862	

*The position according to PPV4 clone C17 GenBank accession number: GQ387500) [6]

Sequence analysis revealed that the JS0910 sample yielded 2 variants, the JS0918 sample yielded 3 variants and the HEN0922 sample yielded 2 variants. These seven PPV4 genomes contained three major ORFs, which included a putative replicase (ORF1), an ORF3 of unknown function and a capsid gene (ORF2). The coding capacity of each ORF was identical to that of the American PPV4 [6]. Phylogenetic analysis showed that these Chinese PPV4 genomes clustered with the American PPV4 genomes. Among the Chinese isolates, the nucleotide sequence identity of ORF1 was 99.1- 99.3%, ORF2 was 99.2-99.8% and ORF3 was 99.2-99.3%. Between the Chinese isolates and the American isolates, nucleotide identity of ORF1 was 99.7-99.8%, ORF2 was 99.2-99.8% and ORF3 was 99.7-99.8%. Among the Chinese isolates, the amino acid identity of ORF1 was 98.9-99.3%, ORF2 was 99-100% and ORF3 was 99.3- 99.6%. Between the Chinese isolates and the American isolates, the amino acid identity of ORF1 was 99.8-100%, ORF2 was 98.5-99% and ORF3 was 99.7-99.9%.

Previous work demonstrated that head-to-tail circular or concatameric PPV4 genomes exist in tissues of the infected animals [6]. Primers designed specifically to detect head-to-tail covalently linked sequences used in the previous study were employed to determined the head-to-tail junction sequences of the seven PPV4 genomes obtained. PCR experiments yielded head-to-tail products of four difference sizes. After sequence determination and assembly, it was noted that the sizes of the seven genomes ranged from 5400 to5644 nucleotides (nt) (GenBank accession numbers: GU978964-GU978968, HM031134-HM031135). In comparison with the genomes of the American PPV4 (5780-5905 nt), all the Chinese PPV4 genomes examined were smaller and the deleted sequence in each genome was located between nt 5964-595 in the non-coding region. The 5400 nt genome (GU978964, GU978968 and HM031134) was detected in all 3 pigs that came from 2 different provinces and the 5644 nt genome (GU978965 and GU978967) was detected in 2 pigs from the same farm. Interestingly, the nucleotide deletions of the three 5400 nt genomes were identical and the nucleotide deletions of the two 5644 nt genomes were also identical.

In conclusion, the results of this study demonstrated that PPV4 existed in some breeding pig farms in China, probably since 2009. Phylogenetically, the Chinese and American PPV4s are closely related and cluster in the same clade. They share greater than 99% nucleotide sequence identity in all 3 ORFs. The American PPV4 was first identified among swine suffering from an acute onset disease of high mortality in North Carolina, USA during late 2005 [14]. Examination of the farm records showed that the two PPV4-positive Chinese farms had purchased swine from a common source in North Carolina prior to the discovery of PPV4. Since the Chinese and American PPV4s are very similar with respect to nucleotide sequence and deletions in the non-coding region, it is possible that PPV4 was introduced into China through the importation of PPV4-infected pigs from the U.S. This study also confirmed previous study [6] that circular or head-to-tail concatameric PPV4 molecules were present in the the diseased animals. The head-to-tail configured PPV4 genomes are likely generated via circularization of the genome in which the left-end and right-end of the linear genome are covalently linked. It is not surprising that some terminal nucleotides of the linear genome may have been deleted during the circularization process, which is observed in this and previous studies. Similar to that of a *Dependovirus*, adeno-associated virus type 2, the presence of head-to-tail configured molecules in the infected animals suggests that PPV4 may be able to establish persistence in its host [15,16]. The fact that PPV4 was detected in diseased animals with other co-infecting viruses, further investigations are needed to determine the pathogenic capability of PPV4 and surveillance studies are needed to determine whether PPV4 could spread and become a serious emerging swine pathogen.

## Competing interests

The authors declare that they have no competing interests.

## Authors' contributions

LH, SLZ and HBZ obtained samples and extracted viral DNA. LH carried out the PCR and sequencing studies and drafted the manuscript. JXL, AKC and SSY conceived the study and reviewed the manuscript. All authors read and approved the final manuscript.
